# Cognitive processes of scientific problem-solving while using simulations: an expert-novice comparison

**DOI:** 10.3389/fpsyg.2026.1846556

**Published:** 2026-06-17

**Authors:** Yating Zeng, Jiayi Tao

**Affiliations:** 1 College of Chemistry, Chemical Engineering and Materials Science, Soochow University, Suzhou, China; 2 Institute of Curriculum and Instruction, East China Normal University, Shanghai, China

**Keywords:** cognitive processes, experts, novices, problem-solving, scientific problems, simulation

## Abstract

The ability to utilize simulations to address problems is emphasized in current science standards, but learners often struggle with scientific tasks involving simulations. Understanding the gap between beginner and expert scientific problem solvers in simulation settings is useful for helping students to develop their expertise. However, limited studies have investigated the differences in cognitive processes between specialists and novices when they tackle simulation-based scientific tasks. Therefore, in this study, the think-aloud method was adopted to compare the cognitive mechanisms of 12 science teachers (experts) and 12 middle school students (novices) as they used simulations to solve scientific problems. The results showed that the experts demonstrated greater cognitive engagement than the novices. Specifically, the experts exhibited a more comprehensive understanding of the tasks. The novices typically ran the simulations blindly and rarely recorded the specific details of the scientific phenomena. The experts also engaged in more logical reasoning, in-depth questioning, and rational decision-making, whereas the novices were more inclined to rely on their intuition and gave hasty responses.

## 1 Introduction

A simulation is a computer program that represents a real process or phenomenon (Fang and Guo, 2023; Wen et al., 2020). Nowadays, simulation has become an indispensable tool for scientists to solve problems. It can model nature (Calogiuri et al., 2023), serve as a functional equivalent for authentic experiments (Reyes et al., 2024), and accelerate scientific discovery (Shinde et al., 2021), thereby greatly improving the efficiency of scientific research (Wang et al., 2024). In science education, simulation has also attracted increasing attention because of its significant potential as a teaching and learning tool, as well as its merits in terms of speed, safety, repeatability, and low cost (Chiou et al., 2019; Dunnagan et al., 2020). Recently, many countries have put an emphasis on integrating simulations into science classrooms and required students to use simulations to complete scientific tasks [Australian Curriculum, Assessment and Reporting Authority, 2022; Ministry of Education, 2023; Next Generation Science Standards (NGSS) Lead States, 2013]. Therefore, a growing body of research has raised concerns about students’ scientific problem-solving performance while utilizing simulations (Andrews-Todd and Forsyth, 2020; Chen et al., 2024).

Studies have demonstrated that students find it challenging to acquire proficiency in utilizing simulations to solve scientific problems (Jeong et al., 2022). Specifically, they have difficulties conducting simulation experiments (de las Heras et al., 2021), employing effective strategies (Park, 2020), and using higher-order cognitive skills (da Silva and de Vasconcelos, 2022) when confronted with simulation-based scientific tasks. As such, it is necessary and urgent to improve students’ ability to handle scientific problems in simulation contexts. However, there is a lack of clear, effective, and targeted methods to do so (Ouahi et al., 2022), which hinders science teachers’ teaching practices.

Novices cannot become specialists overnight; it takes time and considerable effort (Kneebone, 2020). Hence, research needs to identify the differences between novice and expert scientific problem solvers in simulation settings, and to help learners bridge this gap. Over the past few years, science education scholars have explored the differences between experts and novices in scientific problem-solving (Álvarez et al., 2020; Lenzer et al., 2020) and have found notable variations in many aspects. These include differences in planning (Avena et al., 2021), action efficacy (Álvarez et al., 2020), and metacognition skills (Dulger and Ogan-Bekiroglu, 2025). Yet, to date, only a few studies have investigated the distinctions between specialists and beginners in using simulations to tackle scientific tasks. Thus, it is unclear how novices and professionals differ cognitively when handling scientific problems with simulations, as well as how this ability develops.

A comparative study of the cognitive mechanisms employed by novices and experts is a crucial step in identifying the skills that enable the effective use of simulations to address scientific problems. Therefore, this paper aims to examine the similarities and differences in mental processes between novices and experts. The results may shed light on how, where, and when learners differ from specialists, and assist students in moving toward more expert-like performance. This research may also help educators develop instructional interventions for teaching simulation-based scientific problem-solving skills grounded in professionals’ knowledge, expertise, and strategies.

## 2 Research questions

The purpose of this research is to compare the cognitive processes of novices (students) and experts (teachers) as they utilize simulations to solve scientific problems. The research questions are as follows:

(1) What are the cognitive characteristics of experts and novices while solving scientific problems in the context of simulations?
(2) Are there significant differences in cognitive elements or processes between experts and novices when approaching simulation-based scientific problems?

## 3 Literature review

### 3.1 Scientific problem-solving

Scientific problem-solving refers to the capacity to apply scientific knowledge and cognitive skills to achieve a desired goal (Scherer and Beckmann, 2014). According to Zhao et al. (2023), scientific problem-solving encompasses designing, scientific reasoning, and scientific explanation. Tschisgale et al. (2025) have delineated the steps students take to complete physics problems, such as assumptions, hypotheses, concept applications, meta-descriptions, and quantitative manipulation. Nikolić and Antonijević (2024) discovered that the primary actions in addressing biological tasks include planning and analysis, solution generation, evaluation, and discussion.

Different tasks require distinct knowledge, skills, and methods (Ryu et al., 2023; Shvartsman and Shaul, 2023), which may result in varied understandings of scientific problem-solving. For example, well-defined scientific problems follow a clear routine, while ill-defined scientific problems call for multiple and divergent solution paths (Lien et al., 2020). Textbook problems in science classes typically focus on using the correct formula and standardized procedures (Bakken and Andersson-Bakken, 2021; Berge et al., 2020), whereas authentic scientific tasks necessitate multivariable reasoning, claim-evidence coordination, and argumentation (Lesperance and Kuhn, 2023). Basic scientific problems may be solved with little expertise, whilst complicated scientific problems demand extensive skills (Zhao et al., 2023). Static scientific problems provide all the information needed from the outset, yet dynamic scientific problems involve interaction between problem solvers and systems to obtain new information (Wicaksono and Korom, 2025).

### 3.2 Scientific problem-solving in simulation contexts

Simulation contributes greatly to students’ scientific problem-solving activities. It offers more real-world contexts (Lindfors et al., 2020), concretizes abstract and complex scientific phenomena (Li et al., 2024), and dynamically visualizes underrepresented scientific concepts (Martinez et al., 2021). Simulation-based scientific tasks can also enrich the opportunities and means for students to demonstrate their skills (Pang et al., 2025). Thus, these tasks provide a more realistic reflection of problem-solving practice, shed additional light on the reasons behind failed problem-solving attempts, help identify absent sub-abilities, and better distinguish learners who are unable to complete specific tasks (Ulitzsch et al., 2022).

Simulations are becoming a popular subject in the field of scientific problem-solving. For instance, an electrochemistry simulation created by Chen et al. (2024) enabled students to examine and analyze solutions at both macro- and microscopic scales. Andrews-Todd and Forsyth (2020) designed an electrical device simulation that allowed users to adjust the resistor to get the required voltage. Lindfors et al. (2020) developed a simulation toolbox in which individuals could construct and explore a seesaw using various materials of different shapes to achieve balance.

Researchers have explored the cognitive mechanisms involved in scientific problem-solving in simulation scenarios. Teig (2024) asserted that simulation-based scientific problem-solving involves multiple steps, including running simulations, implementing strategies, conducting tests, and providing answers. Falloon (2020) claimed that learners engaged in comprehending, remembering, assessing, contrasting, and analyzing when approaching electricity tasks. After synthesizing prior STEM problem-solving frameworks and integrating computer science features, Priemer et al. (2020) proposed 13 cognitive processes for STEM problem-solving with computer technology. These included recognizing a problem, conducting experiments, observing, organizing data, making inferences, and communicating results.

### 3.3 Differences between expert and novice groups

In general, experts can be defined as individuals who have received formal education and have extensive experience in a certain field; novices can be defined as those who possess limited training or experience in a given field (Qi et al., 2024). When confronted with problems, specialists demonstrate exceptional performance, while novices struggle to handle tasks effectively (Lieberei et al., 2023). In the realm of science education, many researchers consider science instructors as experts due to their professional knowledge and skills (Chang, 2022; Tal et al., 2021). Furthermore, taking into account diverse educational backgrounds and teaching duration, some scholars have suggested that teachers with at least five to ten years of teaching experience can be regarded as experts (Zhao et al., 2022). Additionally, academic and research professionals, such as scientists and PhD holders in science education, are often viewed as authorities (Goldwater et al., 2021; Osborne and Allchin, 2024). Middle school or undergraduate university students are seen as beginners (Lieberei et al., 2023; Pande and Chandrasekharan, 2022).

Numerous studies have demonstrated that experts and novices approach scientific problems with different cognitive mechanisms (Álvarez et al., 2020; Dulger and Ogan-Bekiroglu, 2025). For instance, specialists are able to identify the central idea, whereas beginners often concentrate on surface details (Dai et al., 2019). While carrying out scientific tasks, experts develop more comprehensive plans, apply more knowledge, use more resources, implement more effective methods, take fewer steps, generate more alternative solutions, give higher-quality responses, and ultimately handle problems more accurately compared to novices (Lenzer et al., 2020; Price et al., 2022). In addition, experts exhibit higher levels of metacognitive skills, which facilitate superior planning, monitoring, regulation, and evaluation of their own cognition when addressing scientific problems (Maries and Singh, 2023).

In computer simulation situations, researchers have contrasted the performance of successful and unsuccessful problem solvers (Parno et al., 2021). Wen et al. (2018) found that students who successfully handled simulated tasks often connected the contexts with scientific terms, whereas those who failed relied only on intuitive knowledge. Chang et al. (2017) indicated that the students who perform well in kinematics problems in simulation settings mainly apply analytical reasoning strategies, while those who perform poorly primarily employ trial-and-error strategies. Lee et al. (2019) discovered that professionals outperformed students in accuracy, speed, and systematicity of approaches in a medical simulation scenario. Nevertheless, few research studies have looked at the cognitive differences between specialists and beginners when using simulations to tackle scientific problems.

To sum up, although existing studies have provided a solid basis for comprehending expert-novice differences in scientific problem-solving, research on the cognitive mechanism differences between professionals and beginners when solving scientific problems within simulation environments is limited, and prior studies have focused predominantly on scientific tasks in a specific domain rather than covering a broader range of subjects. Therefore, this study aims to compare the cognitive processes employed by experts and novices in simulation-based scientific tasks and to identify the key cognitive components. By doing so it seeks to advance understanding of this ability, help students (novices) to overcome obstacles, and improve science learning in simulation environments.

## 4 Methods

### 4.1 Design

The think-aloud method involves the verbalization of thoughts while solving a problem (Yang and Zhang, 2023). It is particularly suitable for uncovering the psychological mechanisms of problem-solving, which are not easily captured by other techniques (Blech et al., 2020). In recent years, think-aloud protocols have been widely used to analyze expert/novice differences in cognition (Pilous et al., 2023; Stahnke and Gegenfurtner, 2025). The think-aloud approach can be categorized into concurrent verbalization, which involves reporting thoughts verbally during performing tasks, or retrospective verbalization, which requires individuals to recall their previous steps after completing tasks (Fan et al., 2023; Prokop et al., 2020). Generally, concurrent verbal reports are more reliable than retrospective ones because the latter rely on participants’ memory, which might not accurately reflect the actual problem-solving processes (Padilla and Leighton, 2017). Additionally, participants may add, remove, or alter their mental processes in retrospective reports (Leighton, 2017). Therefore, this study employed the concurrent think-aloud technique to examine the cognitive mechanisms of experts and novices, aiming to reveal the qualitative and quantitative differences between the two groups while using simulations to deal with scientific problems.

### 4.2 Participants

Education level and teaching experience are often used as criteria in science education to classify experts and novices (Lieberei et al., 2023; Pande and Chandrasekharan, 2022). Thus, in this study, purposive and convenience sampling were employed to select 12 experts (8 female and 4 male) and 12 novices (6 female and 6 male). The experts were science teachers with master’s degrees in science education and over five years of teaching experience. The novices were seventh, eighth, and ninth graders from a rural middle school in China, including high, medium, and low academic achievers. As 12 verbal reports are sufficient for providing information on problem-solving behavior (Guest et al., 2020), the study sample size is adequate. Participants volunteered for this study and provided informed consent.

### 4.3 Tasks and procedure

**Figure 1:**
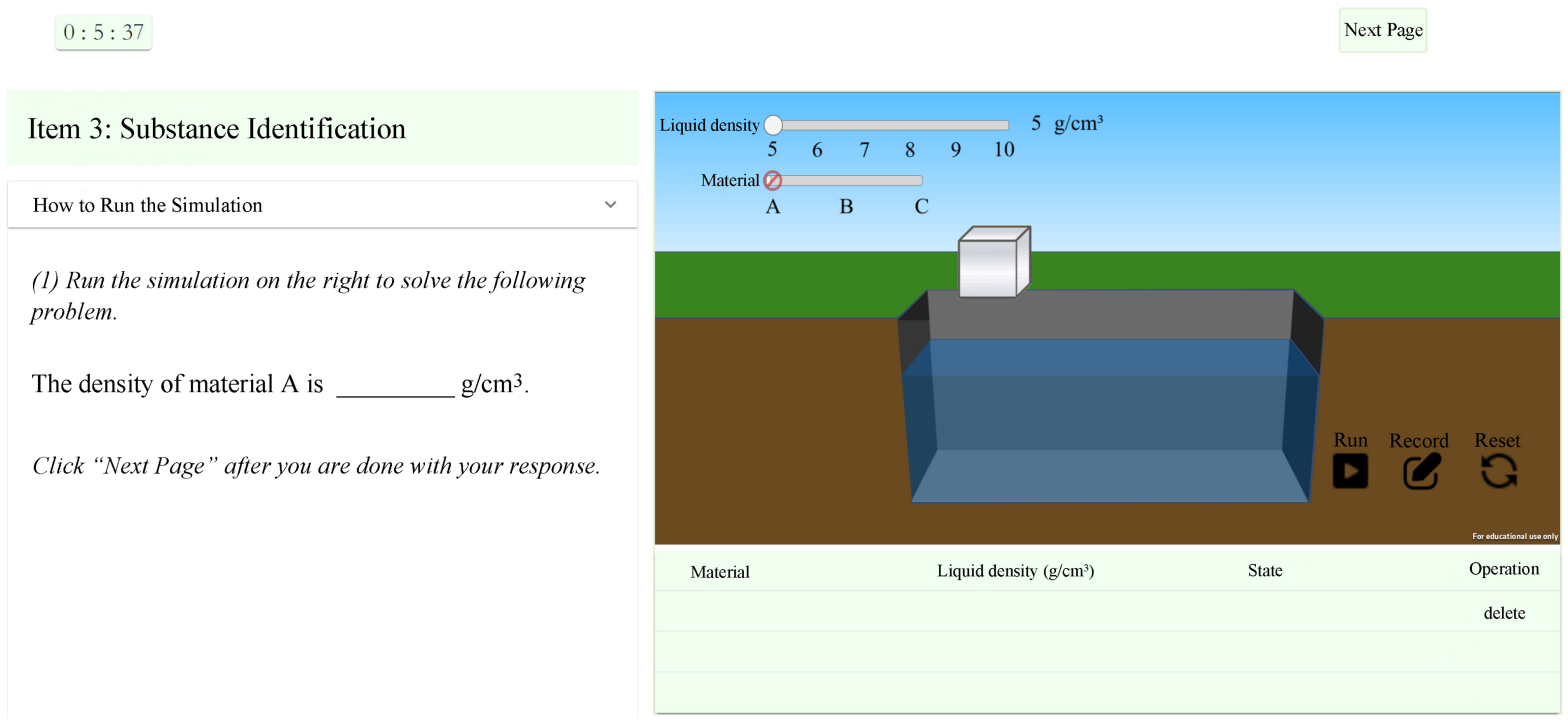
Item 3: substance identification. PhET (2025). License: CC BY-NC 4.0. It permits free use for non-commercial purposes. Source: https://phet.colorado.edu/en/simulations/buoyancy-basics.

With reference to PISA (OECD, 2013, 2017) and NAEP (National Assessment Governing Board, 2009), four items were designed and embedded into three topics: car braking, substance identification, and soil composition. The tasks were derived from real-life situations rather than textbook exercises or exam problems. The requisite scientific knowledge was limited to ensure that participants would understand the problems and to avoid potential test unfairness due to subjects’ prior knowledge. Each item had a matching computer simulation that simplified and idealized the actual world. Specifically, the items omitted irrelevant details and minimized the need for computer skills, thus allowing participants to explore the tasks quickly and efficiently with only a few operations. The simulations were developed based on Physics Education Technology (PhET) interactive simulations (PhET, 2025) and NAEP (National Assessment Governing Board, 2009). The tasks and simulations were integrated into a website, and all items had the same interface. To guarantee comparability, all items were reviewed by three experts in science education, who confirmed that the items were consistent in design, requirements, and complexity. Figure 1 shows an example item that demonstrates the task features and website design.

In October 2025, participants completed the tasks on their own during individual meetings with the researchers. Teachers used their personal computers to address problems, while the students solved problems using computers provided by the researchers. Participants were prohibited from using reference books and other online resources. Before the think-aloud sessions, the researchers explained the study to the participants and obtained their consent. Next, the participants received five minutes of instruction to ensure they were proficient in operating computers and simulations, as well as in verbalizing their thoughts as required. Finally, the participants independently performed the tasks while speaking aloud. If participants remained silent for a few seconds, the researchers verbally reminded them to report their thoughts. There was no time limit for the testing. In the problem-solving procedures, the researchers did not evaluate participants’ responses, and the interference was minimized.

### 4.4 Data collection and analysis

This study used the think-aloud method to collect data. While participants employed simulations to solve scientific problems, their verbalizations were obtained through audio recordings. Participants’ behaviors were also captured by a computer screen recording software to track and verify their cognition.

In accordance with Yang’s (2003) recommendations, participants’ think-aloud protocols were transcribed, segmented, and coded. First, the audio recordings (totaling 5 h and 6 min) were transcribed verbatim into text files, comprising 96 verbal reports from novices and experts. Second, the texts were divided into sections based on cognitive changes rather than following a fixed time frame. Third, each section was coded using a coding scheme (Table 1) adapted from Zeng et al. (2025). Two master’s students majoring in science education served as coders and independently coded the protocols after training. Any discrepancies in the codes were discussed and resolved. The Cohen’s kappa statistic was 0.90, indicating perfect interrater reliability (Landis and Koch, 1977).

**Table 1:** Coding scheme.

Dimension	Code	Definition
Identifying and extracting	Reading	Participants look over the information in the problems.
	Understanding	Participants grasp the information in the problems.
	Associating	Participants think about things that are linked.
Designing and investigating	Planning	Participants make arrangements to tackle problems.
	Exploring	Participants operate the simulations blindly.
	Experimenting	Participants deliberately manipulate the simulations to carry out scientific experiments.
	Observing	Participants see the occurrence presented in simulations.
	Recording	Participants record information presented in simulations.
Analyzing and arguing	Comparing	Participants contrast different things.
	Pondering	Participants contemplate something.
	Reasoning	Participants engage in logical analysis to formulate inferences from proof.
	Guessing	Participants express ideas with no proof.
	Judging	Participants make judgments to approve or reject something.
	Calculating	Participants execute mathematical calculations.
Monitoring and evaluating	Being confused	Participants have difficulty comprehending something.
	Recalling	Participants recall past occurrences.
	Revising	Participants modify their previous ideas or responses.
	Introspecting	Participants reflect on their ideas.
Summarizing and clarifying	Concluding	Participants determine their answers.
	Explaining	Participants explain their answers.

To examine whether novices and experts emphasize cognitive components and stages differently, the frequency and proportion of each code in the think-aloud protocols were calculated to analyze their use during the process of solving simulation-based scientific problems. Considering that cognitive engagement is possibly a function of duration, the time both groups spent on the tasks was also measured. Due to the small sample size and the non-normal distribution of the data, the Mann–Whitney test is appropriate for determining group differences (Chicco et al., 2025; Dehaene et al., 2021). Thus, this study employed the Mann–Whitney test to quantitatively compare the cognitive processes of experts and novices. The statistical analysis was conducted using SPSS 27.0.

## 5 Results

This study measured the frequency and proportion of each cognitive phase and component used by the experts and novices when employing simulations to tackle scientific problems; the results are shown in Table 2.

**Table 2:** The frequency and proportion of cognitive phases and components used by experts and novices.

Cognitive phases	Cognitive components	Novices		Experts	
		Frequency	Proportion	Frequency	Proportion
**Identifying and extracting**		**59**	**0.15**	**96**	**0.13**
	Reading	57	0.14	68	0.09
	Understanding	0	0.00	26	0.04
	Associating	2	0.01	2	0.00
**Designing and investigating**		**171**	**0.43**	**356**	**0.48**
	Planning	22	0.06	31	0.04
	Exploring	78	0.20	44	0.06
	Experimenting	6	0.02	108	0.15
	Observing	55	0.14	137	0.19
	Recording	10	0.03	36	0.05
**Analyzing and arguing**		**74**	**0.19**	**157**	**0.21**
	Comparing	25	0.06	23	0.03
	Reasoning	5	0.01	36	0.05
	Calculating	2	0.01	2	0.00
	Judging	13	0.03	47	0.06
	Guessing	5	0.01	19	0.03
	Pondering	24	0.06	30	0.04
**Monitoring and evaluating**		**20**	**0.05**	**54**	**0.07**
	Recalling	12	0.03	6	0.01
	Being confused	4	0.01	30	0.04
	Revising	2	0.01	6	0.01
	Introspecting	2	0.01	12	0.02
**Summarizing and clarifying**		**70**	**0.18**	**76**	**0.10**
	Concluding	46	0.12	51	0.07
	Explaining	24	0.06	25	0.03
**Total**		**394**	**1.00**	**739**	**1.00**

The bold values represent the frequency and proportion of cognitive phases (i.e., the sum of frequency and proportion of all cognitive components in each phase) used by experts and novices.

As seen in Table 2, the experts utilized cognitive components approximately twice as frequently as novices overall. The Mann–Whitney test shows significant differences between the two groups (*U* = 18.00, *Z* = −3.118, *p* = 0.002, *r* = −0.64). Given that the experts engaged in many more cognitive activities than the novices, the proportion of each cognitive phase and element may more accurately represent the distribution and differences. Hence, the following results focus the statistical analysis on the proportions of thoughts expressed by participants.

Regarding task duration, the experts averaged 11 min to complete the tasks, whereas the novices averaged 8 min. Nonetheless, the Mann–Whitney test results suggest that the experts did not spend significantly more time on the tasks than the novices, neither in overall task duration nor in time allocation per task. Thus, it can be inferred that in this study, the difference in cognitive engagement was not driven by the duration of the activity.

### 5.1 Identifying and extracting

The identifying and extracting phase was the participants’ initial cognitive step in the simulation-based scientific tasks. During this phase, participants read the contexts, comprehended the problems, and made associations. The Mann–Whitney test was employed to examine the quantitative distinctions between the experts and beginners in this period, and the results revealed no significant difference in terms of the percentage of cognitive activities. Concerning the cognitive components, the experts comprehended the problem more often than the novices, and at a higher proportion (*U* = 12.00, *Z* = −3.876, *p* = 0.000, *r* = −0.79).

A qualitative comparison also demonstrated experts’ efforts in task analysis during the identifying and extracting phase. After receiving each scientific problem, 11 of the experts carefully read the task materials and comprehended the situations. For instance, in the topic “soil composition,” Expert 1 first skimmed the problem: “*Two kinds of soil… peat soil and granular soil… combine these two types of soil… cacti prefer soil with good drainage and permeability… which type of combined soil is suitable for cacti?*” Subsequently, she identified the important information and repeatedly read it, as follows: “*permeability… particles… peat soil… granular soil.*” Similarly, Expert 3 directly extracted the key words and emphasized them: “*I need the soil with good drainage and air permeability.*” Expert 2 expressed her understanding: “*If it has good drainage and air permeability, its water retention will be poor.*” Conversely, all 12 novices read the problem quickly without grasping the task well. For example, Novice 1 stated “*good drainage and permeability*” twice and then wanted to select an answer. Novices 2 and 5 manipulated the simulation immediately after viewing the problem. Novice 10 seemed to understand the task; however, he only said “*it states drainage and permeability*” without providing additional details.

### 5.2 Designing and investigating

In the designing and investigating phase, the experts did not exhibit a significantly bigger percentage of cognitive engagement than the novices. Although the proportions of planning and observing between experts and novices were not significantly different, the experts performed a larger percentage of experimental trials (*U* = 9.50, *Z* = −3.709, *p* = 0.000, *r* = −0.76). They also participated in a higher proportion of data recording (*U* = 41.50, *Z* = −1.969, *p* = 0.049, *r* = −0.40). In contrast, the novices often explored simulations without thinking at a greater rate than the experts (*U* = 8.50, *Z* = −3.673, *p* = 0.000, *r* = −0.75).

Despite the substantial verbal output produced by the experts and novices at this stage, there were discrepancies in their cognitive complexity. During investigations, the experts’ cognitive activities were strategic and deliberate, whilst those of novices were exploratory and heuristic. For instance, in the topic “car braking,” Expert 4 employed the vary-one-thing-at-a-time (VOTAT) strategy, stating: “*First, I have to fix the vehicle’s speed and the weather. I set the speed to 50 (km/h), choose a sunny day, and test it on the cement road.*” She subsequently said: “*Next, I’m going to test the asphalt road. It’s still 50 km/h, on sunny days.*” Finally, she stated, “*test the gravel road, at 50 km/h, on sunny days.*” Expert 8 verbalized: “*Control the variables of speed and weather. I need to see how far it goes. To make the occurrence more visible, I opt for the rainy conditions.*” Expert 12 had a similar statement: “*Select a moderate speed, such as 60 (km/h), and then test its braking distance under three road conditions on sunny days.*” Instead, the novices were not thoughtful while engaging with the simulations, repeating attempts until they succeeded. For example, Novices 7 and 9 did not consider the conditions of velocity and weather; they merely clicked on each parameter sequentially to observe the braking outcomes.

### 5.3 Analyzing and arguing

The experts and novices did not differ considerably in the proportion of cognition during the analyzing and arguing phase. Specially, the experts employed a higher share of reasoning than the novices (*U* = 28.00, *Z* = −2.628, *p* = 0.009, *r* = −0.54).

Specifically, the novices struggled to discern the laws governing scientific phenomena illustrated by simulations and tended to make unfounded conjectures. For instance, Novice 1 saw the words “*good drainage and permeability*” and asserted, “*that’s absolutely the choice with a large amount of …and a small amount of …*” After viewing all the options, she hesitated; nonetheless, she trusted her instincts, saying, “*it should contain a small amount of granular soil.*” Novice 11 noted the three types of roads displayed in the choices and stated without any reason: “*most concrete roads (need caution).*” Novice 3 claimed that “*the cement road ought not to be slippery,*” but did not explain why he believed this to be the case. The experts, on the other hand, were more inclined to give careful thought to the problem, conduct in-depth analyses, and use logical reasoning to identify patterns and relationships, thus forming rational judgments. Expert 2, for example, was skilled at utilizing prior knowledge and experience to support reasoning. Upon noticing that “*peat soil contains nutrients*,” she reasoned that “*it means that its particles are small, (making it) easier to leak water and ventilate. Granular soil lacks nutrients, indicating it is rather loose, and has good drainage capabilities.*” After noting that no water was flowing from a large amount of peat soil within five seconds, Expert 5 inferred that “*it indicates that peat soil has markedly low air permeability and considerable water retention capacity.*” Expert 3 found that the braking distance of cars on cement roads exceeded that on asphalt roads, arguing that “*cement roads are rather slippery*”.

### 5.4 Monitoring and evaluating

There was no disparity between the experts and novices in the proportion of cognition during the monitoring and evaluating phase. Particularly, the experts yielded a greater percentage of confusion than the novices (*U* = 27.50, *Z* = −2.655, *p* = 0.008, *r* = −0.54), with no significant differences observed in other cognitive elements.

To be more specific, the experts kept a close eye on the problem-solving process and quickly identified any issues. For example, when the phenomenon did not match expectations, Expert 8 expressed perplexity: “*0 mL (water comes out)? That is a bit exaggerated, is not it?*” Suddenly realizing his mistakes, Expert 12 remarked: “*Is there another possibility of not adding… right?. There ought to be eight alternatives, right?*” Expert 2 even questioned: “*Do I have to choose the one with the highest flow rate? If the flow rate is very high, the cactus will dry up and die.*” It is clear that the specialists were not only aware of their own cognitive limitations but also carefully considered the essence of each problem. The novices seldom monitored their scientific problem-solving process. Even when some problems emerged, they were unable to sense them. Their doubts mainly stemmed from their inability to comprehend the tasks. For instance, Novice 1 asked: “*Should I choose the phenomena of floating or sinking?*” Similarly, Novice 2 asked: “*Does it need more attention when it’s faster? Or slower?*” Moreover, the experts’ thoughts were more rigorous and deliberate. For instance, Expert 12 initially felt that “*The more granular soil, the better.*” He then immediately revised this as follows: “*No. A small amount of… A large amount of… We will see the results. Do not rush to a conclusion.*” Next, he stated, “*conduct an experiment.*” Expert 7 basically decided the answer, but she reaffirmed her judgment via experiments, saying: “*Wait, I want to see the phenomenon at 9 g/cm^3^.*” Conversely, the novices rarely revised or reflected on their own thoughts except when determining their final responses.

### 5.5 Summarizing and clarifying

During the summarizing and clarifying phase, there was a significant proportional difference between the experts and novices (*U* = 27.50, *Z* = −2.578, *p* = 0.010, *r* = −0.53). Regarding cognitive elements, the experts demonstrated a much higher percentage of concluding (*U* = 28.00, *Z* = −2.554, *p* = 0.011, *r* = −0.52).

In the final step of problem-solving, both the experts and novices provided answers to the problems. The experts’ responses were quite detailed. For instance, Expert 12 said: “*It was found that the cement roads need the most attention to avoid rear-end collisions. The experiments showed that the cement roads had the longest braking distance under the same weather and at the same speed. This helps us to conclude.*” Expert 1 stated: “*According to the information provided in the question, peat soil contains nutrients, but granular soil does not. Therefore, I should combine a large amount of granular soil with a small amount of peat soil for this cactus.*” However, the majority of novices just clicked on the selections or completed the blank fields, making comments such as “*I think…this option*” or “*Choose it*”.

Overall, while using simulations to address scientific problems, the experts demonstrated more cognitive engagement than the novices across the five cognitive phases. Although both the experts and novices underwent a series of procedures, including reading tasks, manipulating simulations, observing phenomena, and providing answers, they differed quantitatively and qualitatively in cognitive elements. The novices ran simulations mindlessly, struggling to understand the tasks and discover the underlying logic of scientific phenomena. In contrast, the experts were capable of comprehending scientific problems, employing scientific strategies to conduct experiments, recording data, forming rationales about the observed phenomena, posing timely questions, and drawing rigorous conclusions.

## 6 Discussion

Students had difficulty addressing scientific problems while using simulations. In order to assist students to develop scientific problem-solving skills in simulated settings, it is essential to identify and bridge the skill gap between novices and experts. However, little research has explored how specialists and beginners solve simulation-based scientific problem-solving. Thus, the purpose of this study was to uncover and compare the cognitive differences between experts and novices when employing simulations to approach scientific tasks.

Consistent with existing studies (Álvarez et al., 2020), the findings show that the experts engaged in a higher number of cognitive activities than the novices when solving simulation-based scientific problems. There were also distinctions between the experts and novices in how the problems were perceived and addressed, which echoes the discoveries of other researchers (Davis et al., 2024; Tóthová and Rusek, 2022).

The results of this research suggest that during the identifying and extracting phase, the experts carefully examined the context of the scientific tasks, whereas most of the novices hastily skimmed over the tasks, lacking awareness and comprehension of the problems. This aligns with the view of prior researchers (Agnello and Focant, 2025; Nelms and Segura-Totten, 2019; Tóthová and Rusek, 2025), who found that specialists allocated time to read relevant materials and had few comprehension difficulties, while beginners were less efficient in identifying important information, which hindered their comprehension of the problems. Thus, the problem-solving theory posits that rich problem representations differentiate specialists from beginners (Jonassen, 2000). Dai et al. (2019) also found that novices often concentrated on superficial features and made a direct connection between contextual characteristics and task outcomes.

Our findings indicate that during the designing and investigating phase, the specialists strategically utilized simulations to carry out experiments, while the novices explored the simulations blindly. This result is similar to that of Zhao et al. (2023), who found that successful problem solvers tended to design and conduct scientific experiments rigorously within simulation settings. Furthermore, when solving scientific problems, the actions of the specialists were effective and structured, whereas those of the novices were unproductive and disorganized (Álvarez et al., 2020; Tschisgale et al., 2025). These differences may be attributed to the experts concentrating on the correct responses, while the novices’ attention was split across many areas of interest (Blinnikova and Ishmuratova, 2018; Tóthová and Rusek, 2025). In addition, the experts prioritized planning over execution (Price et al., 2021), while the novices engaged in far less planning (Burkholder et al., 2020). Another difference relates to recording. This result provides evidence that data recording is crucial to scientific investigations, as the goal of science is to document the process of logic and argument that leads to conclusions (Buchanan, 2016).

During the analyzing and arguing process, the novices and experts differed most on reasoning. This finding is consistent with that of Álvarez et al. (2020), who observed that beginners struggled to use reasoning when tackling scientific problems. It also echoes the research of Anderson and Leinhardt (2002), who discovered that the reasoning process can be used to classify novices’ and experts’ problem-solving in geography. Accordingly, Cheng et al. (2018) concluded that the best predictor of scientific problem-solving skills is reasoning. Additionally, the specialists generally used their knowledge or experience to obtain correct rules, while the beginners mostly depended on memorizing rules and algorithms (Chen et al., 2020). Several studies (Bower et al., 2024; Nelms and Segura-Totten, 2019) have stated that experts and novices process information in various ways, with experts more likely than novices to analyze and evaluate scientific information, resulting in divergent judgments about the same data.

The results revealed that during the monitoring and evaluation phase, the experts exhibited more metacognition than the novices. It demonstrated that specialists were able to question the scientific problems and reflect on their problem-solving process. This finding aligns with previous research (Álvarez et al., 2020; Persky and Robinson, 2017). A potential explanation for this disparity between experts and novices is that professionals are more cautious than beginners in addressing scientific problems (Balta and Asikainen, 2019). In Dulger and Ogan-Bekiroglu’s (2025) study, the novices showed weakness in metacognitive knowledge and monitoring, which are essential skills for developing effective solutions to scientific problems. So Randles et al. (2018) posited that one trait that can distinguish a novice from an expert problem solver is the ability to become reflective when solving scientific problems.

The novices and experts differed throughout the summarizing and clarifying stage, with the specialists clearly expressing their conclusions and justifications, while the beginners often made decisions simplistically and rapidly. Other researchers (Price et al., 2022) also found that experts concentrated on the key details to verify and check their solutions, whereas students provided all available information with their final responses. This may be because novices only focus on solving problems immediately, while experts arrive at conclusions after grasping the nature of the problems (Kim and Klassen, 2018).

## 7 Conclusions and limitations

This research examined the disparities in the cognitive processes used by experts and novices when addressing simulation-based scientific tasks. Quantitative and qualitative analyses of the think-aloud reports revealed that the specialists engaged in more cognitive activities than the novices while using simulations to tackle scientific problems. In particular, the experts demonstrated strong scientific task understanding, strategic simulation manipulation, detailed recording, logical reasoning, rational thinking control, and comprehensively concluding. The novices, on the other hand, lacked problem comprehension, operated simulations at random, made decisions quickly, and were prone to rely on their intuition and personal feelings.

It should be noted that the cognitive phases and components in which the experts and novices differed significantly are crucial to scientific problem-solving in simulation environments. This means that teachers can help learners become more adept at utilizing simulations for scientific tasks by providing practice in key cognitive activities and critical cognitive elements (i.e., understanding, experimenting, recording, and reasoning). For instance, when students observe scientific phenomena in simulations, educators can remind students to collect data, such as variables, parameters, conditions, and outcomes, and use this information to form judgments and draw conclusions. Teachers can also scaffold their reasoning by asking students about the underlying causes behind the scientific phenomena and encouraging them to discern patterns or laws based on evidence instead of intuition. Students can develop their scientific problem-solving skills by emulating and learning explicit cognitive processes from professionals. In addition, the findings may assist researchers in creating instruments for evaluating scientific problem-solving skills in simulation scenarios.

The research has several limitations. First, since scientific problem-solving is domain-specific, it is difficult to generalize the results to other simulation and task types that vary greatly. Second, as the sample size was relatively limited, and the novices were from the same middle school, the findings are not generalizable to different geographical areas and the wider population. Third, although this study attempted to minimize potential bias arising from participants’ prior knowledge, differences in age and level of education may still have introduced confounding variables (e.g., general cognitive development). This suggests that the observed differences might not be solely due to scientific expertise but also because adults have fully developed cognitive faculties, higher reading comprehension levels, stronger reasoning abilities, and greater maturity compared to young adolescents. Additionally, group comparisons could be affected by regional differences or cultural backgrounds. Rural students in China have restricted access to educational resources, and their limited economic and cultural capital may impede their direct experience with specific objects, concepts, terminologies, and technologies, perhaps resulting in a disparity in cognitive processes. Fourth, this study did not investigate the motivations or emotions that may influence the performance of simulation-based scientific problem-solving.

Given the above limitations, future studies could use a larger sample and a more diverse population to capture richer data. The difference between experts and novices concerning their scientific problem-solving processes should be explored in other simulation settings. Further research is needed to control for age by comparing, for example, novice teachers to expert teachers, or university freshmen to graduate students. Other probable sources of bias, such as level of education, region of residence, and cultural factors, should also be considered and addressed in subsequent works to ensure more accurate results. Researchers could also investigate distinction in non-cognitive features (e.g., motivations and feelings) to provide a more comprehensive and deeper understanding of simulation-based scientific problem-solving.

## Data Availability

The original contributions presented in the study are included in the article/supplementary material, further inquiries can be directed to the corresponding author.
